# A Rivest–Shamir–Adleman-Based Robust and Effective Three-Factor User Authentication Protocol for Healthcare Use in Wireless Body Area Networks

**DOI:** 10.3390/s23218992

**Published:** 2023-11-05

**Authors:** Kaijun Liu, Guosheng Xu, Qiang Cao, Chenyu Wang, Jingjing Jia, Yuan Gao, Guoai Xu

**Affiliations:** 1 School of Cyberspace Security, Beijing University of Posts and Telecommunications, Beijing 100876, China; liukaijun@bupt.edu.cn(K.L.); guoshengxu@bupt.edu.cn (G.X.); wangchenyu@bupt.edu.cn (C.W.); jiajingjing@bupt.edu.cn (J.J.); yuan_gao@bupt.edu.cn (Y.G.); xga@hit.edu.cn (G.X.); 2 Key Laboratory of Trustworthy Distributed Computing and Service (MoE), Beijing 100876, China; 3 School of Computer Science and Technology, Harbin Institute of Technology, Shenzhen 518055, China

**Keywords:** authentication, healthcare, Rivest–Shamir–Adleman (RSA), hash function, wireless body area networks (WBANs)

## Abstract

In healthcare, wireless body area networks (WBANs) can be used to constantly collect patient body data and assist in real-time medical services for patients from physicians. In such security- and privacy-critical systems, the user authentication mechanism can be fundamentally expected to prevent illegal access and privacy leakage occurrences issued by hacker intrusion. Currently, a significant quantity of new WBAN-oriented authentication protocols have been designed to verify user identity and ensure that body data are accessed only with a session key. However, those newly published protocols still unavoidably affect session key security and user privacy due to the lack of forward secrecy, mutual authentication, user anonymity, etc. To solve this problem, this paper designs a robust user authentication protocol. By checking the integrity of the message sent by the other party, the communication entity verifies the other party’s identity validity. Compared with existing protocols, the presented protocol enhances security and privacy while maintaining the efficiency of computation.

## 1. Introduction

With the development and maturity of wireless communication technologies, wireless networks have been widely used to obtain specific information; this has a profound impact on how we live, work, and play. It is well known that wireless body area networks (WBANs), as a promising application of wireless networks in healthcare, have attracted significant attention with their foreseeable potential to improve the quality of healthcare services. As defined in the IEEE 802.15.6 standard [1], WBANs are composed of wearable, implantable, and invasive intelligent electronic devices around the human body; currently, most wireless medical devices operate under the 2.4 GHz band [2].

**Figure 1:**
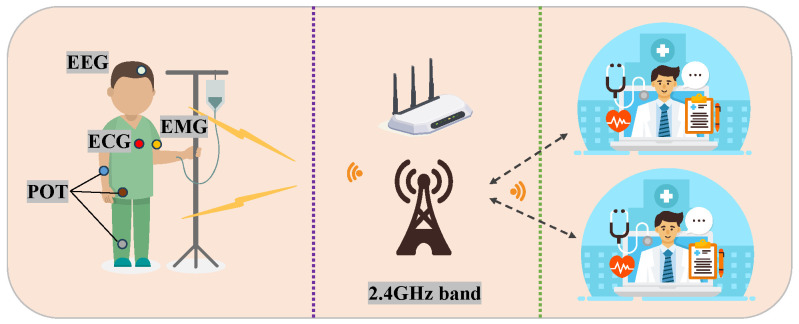
Network model of WBANs.

As shown in Figure 1, electroencephalogram (EEG) sensors can monitor different types of brain waves. Electromyography (EMG) sensors can test muscle activity. Electrocardiogram (ECG) sensors can detect the electrical transmissions of the heart. Pulse oximeter (POT) sensors can measure hemoglobin in the blood. These health facilities, i.e., EEG, EMG, ECG, and POT sensors, have been used to assist physicians in empowering the functions of human gait analysis, postoperative rehabilitation monitoring, sleep quality detection, and respiratory disease prevention [3]; then, they enable physicians to provide timely medical services without geographical limitations.

However, the openness of wireless communication undoubtedly runs the risk of unfettered illegal access, which may, on the one hand, distort professional diagnosis and treatment and, on the other hand, leak patients’ personal vital and sensitive physiological data. With so much real-time data traveling from body area sensor nodes to physicians, just as much attention must be paid to security and privacy issues as vaccine research and the elimination of COVID-19 [4].

**Figure 2:**
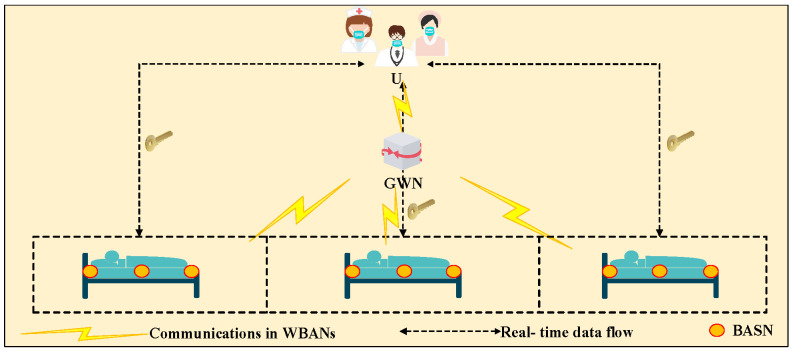
System architecture of WBANs in healthcare.

Luckily, a user authentication mechanism—as a first line of defense for information security that identifies the authenticity of users—is required to protect these key real-time medical data from unauthorized access. As with wireless medical sensor networks (WMSNs) [5], WBANs consist of ad hoc sensor networks, which are continuously carried by patients, connected to their bodies, to sample patient body data. Specifically, the authentication for WBANs involves three indispensable participating entities (shown in Figure 2): a user (U), a gateway node (GWN), and a series of body area sensor nodes (BASNs). The U is often the physician who holds the smart card, the GWN can be a personal digital assistant (PDA), which can be charged, and the BASNs are suitably deployed in patients’ bodies and continuously collect medical data [6]. After the mutual authentication among the three entities, a negotiated session key between the user and BASNs will be generated with the help of the GWN.

However, considering the security threats (such as no forward secrecy for the session key, no user anonymity, and inevitable attacks) in existing WBAN-oriented authentication schemes and the financial expenses of energy-constrained sensor nodes, designing a solution that both strengthens efficiency and security and achieves a good balance between efficiency and security is a challenge [7]. For this challenge, we use the Rivest–Shamir–Adleman (RSA) cryptosystem [8] to only protect the secret values; we do not involve RSA in the computation of session keys to preserve efficiency, and add secret values and the “modulus” operation to enhance security. Then, we consider a three-factor authentication scheme, which can be more suitable for WBANs with limited resources.

### Motivations and Contributions

WBANs, with the guard of the authentication mechanism, can avoid unauthorized access from malicious attackers. However, existing alternatives show deficiency, either in terms of superior performance or robust security. In addition, a greater number of solutions compromise security in favor of higher efficiency. This compromise prompts the design of an efficient user authentication scheme with robust security. Accordingly, three indispensable research works listed below are our research contributions:

(1) The design of a user authentication protocol for WBANs.

Firstly, we show the detailed user authentication system model for WBANs and then give a complete, three-factor (namely smart card, password, and user’s personal biometric information) user authentication protocol, which can obtain mutual authentication from the user, the gateway, and the BASNs. Meanwhile, this protocol offers a user-friendly property whereby the user can locally change or update their password without interacting with the gateway, and can enable BASN deletion/addition at will.

(2) A detailed security analysis for the proposed protocol.

Second, by preserving the user anonymity, a session key is securely established by the user and BASNs. After that, the proposed protocol is demonstrated to be provably secure by the formal security in the random-or-real model. Moreover, a heuristic security analysis has been carried out to show that the proposed protocol can obtain various desirable features and be resistant to all known attacks.

(3) The complete performance comparisons for the proposed scheme.

Third, performance comparisons—covering storage, communication, and computation costs—between our protocol and other existing relevant protocols have been performed. It is clear that the presently proposed protocol can obtain a better balance between efficiency and security than the alternatives.

To give readers a road map of what to expect in the subsequent sections, the rest of this paper is organized as follows. The related works are introduced in Section 2, and preliminary works are shown in Section 3. The designed protocols are detailed in Section 4. In Section 5, a full security analysis is presented for the proposed protocol, including performance evaluation. A final conclusion is made in Section 6.

## 2. Related Work

Kumar et al. [9] were the first to propose an authentication protocol for securing communications in medical healthcare. From then on, a large number of related authentication protocols were developed to enhance the protocol security and performance efficiency [10,11,12,13,14,15,16,17].

In one of the proposed research works, Mo et al. [10] highlighted that common attacks (password guessing attack; desynchronization attack) still threaten the security of the designed protocols. Then, they gave some countermeasures to thwart the vulnerabilities, e.g., using the “modulus” operation to resist the password guessing attack, and making the parameters unchanged to resist desynchronization attacks. Similarly, Khan et al. [11] examined and pointed out that their analyzed scheme cannot guarantee the security of passwords and had no forward secrecy of the session key and no user anonymity [11]. Following this, based on the achievement of [11], Khan et al. [12] offered an improved user authentication scheme for healthcare applications, and they showed that their scheme can be more robust than other analyzed schemes.

In 2013, He et al. [13] listed seven functionality requirements of the authentication solution and then presented a robust anonymous authentication protocol for healthcare. Lately, the work in [14] found that He et al.’s scheme had an incorrect authentication and session key agreement phase and that there was no wrong password detection mechanism. Then, they introduced biometric information as the third authentication factor and designed a three-factor authentication solution to remove the drawback of the scheme in [13]. However, Das et al. [15] showed that the scheme presented in [14] could not resist the privileged-insider attack or the sensor node capture attack; then, they developed a more secure biometric-based user authentication scheme. This was more secure than the schemes proposed in [13,14], in which the additional BAN logic AVISPA tool had been used to prove the security of their proposed scheme.

Focusing on securing the communication in wireless healthcare sensor networks, the authors of [16] presented a three-factor user authentication and key agreement protocol. Their work considered a more user-friendly property for re-registration to accommodate cases in which a user may have lost their smart card or their smart card was stolen. Aiming to resolve security issues in telecare medicine information systems, Ostad et al. [17] developed an enhanced, anonymous, and unlinkable user authentication and key agreement protocol; here, the protocol can provide perfect forward secrecy, patient anonymity, and unlinkability. However, the security of the password could not be preserved, because, in their protocol, the patient directly submitted the bare information $OPW_{p}$ to the server; this can enable the server to easily guess their password.

Afterwards, for securing the authentication in WBANs, Zhang et al. [18] proposed a privacy-preserving authentication protocol between the user and the telecare medical server, and the session key can be used for forward secrecy by using the chaotic map; however, their scheme inevitably suffered from user identity leakage and password guessing attacks (i.e., the insider attacker guesses $PW_{i}^{*}$ and then checks whether $PW_{i}^{*}=W_{i}⊕ID_{i}⊕Z_{i}⊕h\left(ID_{i}⊕PW_{i}^{*}⊕V_{i}⊕h\left(ID_{i}⊕PW_{i}^{*}\right)\right)$.

Very interestingly, for authentication with access control in medical settings, Soumya Banerjee et al. [19] designed a user authentication and session key exchange protocol, in which any physician with their medical department and professional title will only obtain mutual authentication from the designated sensing devices, while anyone who has been revoked or whose authentication credential is overdue cannot obtain authentication anymore. However, we find that their scheme will be under threat due to an absence of forward secrecy, leaving it vulnerable to the password guessing attack and the node capture attack [20].

In a cloud-of-things-centered wearable device monitoring system, based on the Chinese Remainder Theorem [21] (CRT), the research work in [22] presented a secure user authentication with access control scheme whose functionality is similar to the work in [19], in which an observation highlights that, for the forward secrecy, they adopted a principle wherein the long-term key does not need to be involved in constituting the session key. However, significantly more storage resources are consumed in their scheme, which should be optimized.

Using the well-known RSA-based cryptosystem, Dharminder et al. [23] propounded an RSA-based authentication protocol for two communication entities: the user and the telecare server, whereas security flaws—including vulnerabilities to the password-guessing attack, the absence of forward secrecy, and the absence of user anonymity—should be mitigated.

To solve the session key’s forward secrecy, Mahdi Fotouhi et al. [24] offered a robust WBAN-oriented authentication scheme. Their scheme can obtain perfect forward secrecy (PFS) by adopting the secret and updated dynamic authentication credential (DAC) parameters [25], whereas considering the adversary as an administrator of the gateway means that their scheme is defeated in internal anonymity [26]; meanwhile, a very large amount of storage resources are consumed to store indispensable information, including the user data and hundreds or thousands of medical devices.

In contrast to the general centralized system architecture, to mitigate the single point of failure and the trust problem, a blockchain-based authentication scheme [27] was proposed. In their scheme, through the certificate-free-authentication key agreement, each PDA from the WBANs acquires authentication through blockchain nodes, and then these security-critical medical data will be stored in the blockchain. Through blind signature technology, each node can verify the authenticity of an entity that wants to query the medical data.

In 2021, Masud et al. [28] used the physical unclonable function (PUF) [29] and designed a robust user authentication and key establishment scheme, where their scheme can attain perfect anonymity through a very large challenge–response pair. However, to preserve forward secrecy, all entities in the scheme must run the operation of verification at least twice during the authentication phase.

To obtain a superior authentication performance, a lightweight WBAN-oriented scheme [30] was proposed; in this process, a session key is established in the sensor node and the Hub node. However, a deficiency was shown in that there was no mutual authentication among the three entities (access point, hub node, and sensor node); this creates obstacles when encountering real-world applications. Then, Xie et al. of [31] analyzed that the protocol [30] cannot resist a stolen-verifier attack and has no perfect forward secrecy. Then, a robust patient monitoring authentication scheme based on elliptic curve cryptography (ECC) [32] was proposed, and the formal security proof demonstrates the security of their scheme. However, it still lacks mutual authentication between the relay node and sensor node.

To obtain mutual authentication among all entities, Narwal et al. in [33] demonstrated mutual authentication among three entities (sensor node, mid node, and chief node) in their paper. However, their scheme still weakens the session key’s forward secrecy and resistance against the node capture attack. Focusing on the WBAN scenario, the authors of [34] offered a mutual authentication protocol for securing the communication between body sensor units (BSUs) and administrator (Adm); here, the session key can be used for forward secrecy. However, the anonymity of identity should be improved if one considers the GWN as an insider attacker.

In summary, existing alternatives show deficiencies, either in terms of superior performance or robust security. Furthermore, a greater number of solutions compromise security in favor of higher efficiency. It is necessary to design an authentication protocol with higher efficiency and while preserving robust security.

## 3. Preliminaries

### 3.1. System Model

**Figure 3:**
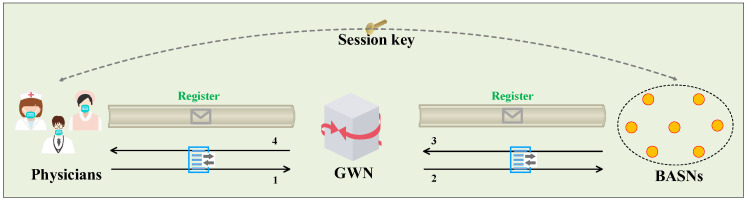
System model in the proposed scheme.

The system model shown in Figure 3 consists of three entities: the physicians, the gateway node (GWN), and a series of body area sensor nodes (BASNs). Furthermore, the GWN computes and then transmits messages between physicians and BASNs; BASNs are constantly carried on a patient’s body and collect real-time data from the body. Furthermore, the physicians comprise entities who directly access the data from the BASNs to monitor patients and then provide timely medical service.

Note that, in Figure 3, information packets in the secure channel are transmitted during the registration phase, and information packets in the public channel from **number 1 to number 4** are transmitted during the authentication and key agreement phase. We then introduced the implementation of authentication and key agreement among the three entities. In the beginning, the GWN initiates the authentication system and then generates a long-term key, a secret key value, and other public parameters. Then, when a physician (denoted by $U_{i}$) registers in the GWN through the secure channel, they send the registration request to the GWN, and then the GWN sends a smart card securely to the $U_{i}$ (information packets shown on the left). For the registration of the BASNs, which also occurs through the secure channel, these nodes only need to submit their identities to the GWN, and then they can receive the identity-related secret values from the GWN’s calculations (information packets shown on the right).

In the following authentication, firstly, the $U_{i}$ submits a login request to the GWN (**number 1**); the GWN then verifies the identity of the $U_{i}$ based on the login request and sends the verification message to the BASNs (**number 2**). After receiving the verification message, the BASNs first verify the GWN and calculate a message which consists of a session key and relevant authentication parameters, and then send this message to the GWN (**number 3**). After obtaining the message from the BASNs, the GWN verifies the BASNs and sends the message with the newly embedded session key to the $U_{i}$. Finally, the $U_{i}$ authenticates the GWN, and obtains key parameters from the received message; then, it recomputes the session key (**number 4**).

### 3.2. RSA Cryptosystem

As a public key cryptography, the Rivest–Shamir–Adleman (RSA) encryption and decryption algorithm [8], based on the hardness problem of a large-number factorization problem, is described below with an example of a message sender ***S*** sending a message ***m*** to a message receiver ***R***.

- **Initiation**: Message receiver ***R*** selects two large prime numbers p,q, computes n=p×q and Euler’s totient function of *n*, i.e., φ(n)=(p−1)×(q−1). Then, ***R*** chooses an integer *e* meeting gcd(e,φ(n))=1, and computes $d\equiv e^{-1}$ (mod φ(n)). As a result, ***R*** is public (e,n) and keeps the private key *d* secret.
- **Encryption**: Message sender ***S*** takes a message ***m*** and computes an encryption $c=m^{e}$ mod *n* with ***R***’s public key *e*. Then, ***S*** sends the cipher *c* to ***R***.
- **Decryption**: Upon receiving the cipher *c*, ***R*** decrypts $m=c^{d}$ mod *n* with their own private key *d*.

### 3.3. Threat Model

The Dolev–Yao model [35]—which depicts the adversary’s capacity—has been widely applied to analyze the security of the authentication protocol. Now, the newest research work [20] further summed up the capabilities of adversaries aiming to fully assess the proposed schemes. Then, in this more sophisticated threat model, an attacker A can be described to have seven capacities (**A-**), as outlined below:

- **(A-1)** A can fully control the open channel and then intercept, modify, insert, and delete any messages transmitted in the open channel.
- **(A-2)** A can enumerate all items offline in the Cartesian product of the identity space and the password space $D_{id}\times D_{pw}$ within the polynomial time.
- **(A-3)** To a three-factor user authentication scheme, A can compromise the following two of three authentication factors: (a) user’s password; (b) data in the smart card; and (c) user’s biometric information.
- **(A-4)** A can obtain previous session keys established between the physician (user) and body area sensor node (BASN).
- **(A-5)** A can grasp GWN’s secret key when we consider the system’s eventual failure.
- **(A-6)** A can break some BASNs, i.e., extracting the sensitive data stored therein, and control the broken BASN to join the next newly communication of GWN, other users, and body area sensor nodes.
- **(A-7)** A may register as a legitimate user or as the administrator of the GWN, only when the security of the user’s password is assessed.

## 4. The Proposed Protocol

In this part, the following indispensable phases covering the user/body area sensor node registration, user/body area sensor node mutual authentication, password change, and body area sensor node deletion and addition constitute a robust RSA-based three-factor user authentication and key agreement protocol. Furthermore, we reinforce the session key’s security and user anonymity from the points below:

1. To achieve forward secrecy, the session key will be computed from the secret values of user and BASNs, rather than the general GWN’s long-term key *x*. Although the adversary grasps *x*, they cannot corrupt the session key. Furthermore, the RSA-based encryption and decryption algorithm will only be used to protect secret values of user and BASNs, but not involving the computation of session key to preserve the efficiency.
2. To preserve user anonymity, in the registration phase, the user only submits the hashed value $A_{0}$ to GWN, and no real identity information has been exposed to adversary (i.e., identity protection); on the other hand, in the verification phase, the dynamic pseudo identity $PID_{i}$ will be allocated to the user. The randomness of $PID_{i}$ confuses the adversary to decide whether two sessions are from the same user (i.e., untraceability).

To facilitate an understanding of the proposed protocol for readers, the notations used in this paper are explained in Table 1.

Next, we provide a detailed description of the proposed protocol.

### 4.1. System Setup Phase Run by GWN

Given a security parameter *n*, the GWN chooses a long-term key $x\in {\left\{0,1\right\}}^{n}$ and keeps *x* secret.

### 4.2. Registration Phase of User and BASN

The registration phase enables the user and the body area sensor nodes (BASNs) to finish the registration of related identity information in the terminal of the GWM; meanwhile, the user and the BASNs receive feedback from the GWN to be ready for future identity authentication. Specifically, two parts are involved—one for the body area BASN and another one for the user/physician ($U_{i}$).

For the registration of each body area a sensor node called $MS_{j}$, $MS_{j}$ sends its identity $MIS_{j}$ to the GWN by the secure channel. Upon receiving the registration request from $MS_{j}$, the GWN computes $x_{j}=h(MIS_{j}\left||x\right)$ and then feeds back $x_{j}$ to $MS_{j}$ also by the secure channel. Meanwhile, the GWN publishes a revocation list $L_{revoke}$ which will store the identity of deleted sensor nodes.

For the new user, $U_{i}$, to register, they need to follow the three following steps with the help of the GWN.

- **Step 1.** $U_{i}⟹GWN:A_{0}$. $U_{i}$ chooses their own $ID_{i},PW_{i}$ and a random value *r*, and computes $HPW_{i}=h(ID_{i}||PW_{i})$ mod $n_{0},A_{0}=HPW_{i}⊕r$. Then, the $U_{i}$ sends the value $A_{0}$ to the GWN through the secure channel.
- **Step 2.** $GWN⟹U_{i}:\left\{PID_{i},BKG\left(\cdot \right),A_{1},Cou\right\}$. Through the received $A_{0}$, GWN firstly generates a pseudo-identifier, $PID_{i}$, computes $V_{i}=h(PID_{i}||x),A_{1}=V_{i}⊕A_{0}$, and then injects the values $PID_{i},BKG\left(\cdot \right),A_{1},Cou$ to the smart card, in which the “Cou” means the maximal times (such as 3). This allows the user to try to login using the smart card if they forget the right password. Lastly, GWN also feeds back the smart card to $U_{i}$ by the secure channel.
- **Step 3.** After obtaining the smart card from the GWN, the $U_{i}$ inputs their biometric information $bio_{i}$ into the smart card, and the smart card further computes $V_{i}=A_{0}⊕A_{1},V_{ii}=BKG\left(bio_{i}\right)$ and $A_{2}=h(ID_{i}||PW_{i}\left|\right|V_{i}\left|\right|V_{ii})$ mod $n_{0}$. In the end, the smart card updates $A_{1}=V_{i}⊕HPW_{i}$ and stores $<PID_{i},BKG\left(\cdot \right),A_{1},A_{2},Cou>$.

### 4.3. User Login Phase

In the login phase, the $U_{i}$ needs to be verified by the smart card. Once the smart card verifies the $U_{i}$’s legitimacy, the $U_{i}$ successfully logs in using the smart card, and the smart card generates an authentication request for the $U_{i}$. Finally, the smart card transmits this request packet to the GWN. Specifically, the $U_{i}$ enters ($ID_{i}^{*}$,$PW_{i}^{*}$) and their own biometric information $bio_{i}^{*}$, then the smart card computes $HPW_{i}^{*}=h(ID_{i}^{*}||PW_{i}^{*})$ mod $n_{0},V_{i}^{*}=HPW_{i}^{*}⊕A_{1},V_{ii}^{*}=BKG\left(bio_{i}^{*}\right)$, and a verifier $A_{2}^{*}=h(ID_{i}^{*}||PW_{i}^{*}\left|\right|V_{i}^{*}\left|\right|V_{ii}^{*})$ mod $n_{0}$, and checks whether $A_{2}^{*}=A_{2}$; here, $A_{2}$ has been stored in the smart card during the registration phase (step 3). If $A_{2}^{*}\neq A_{2}$, then the smart card terminates this session and sets Cou=Cou+1 at the same time. If Cou exceeds a certain value, such as 3, then this smart card is directly suspended until the physician $U_{i}$ re-registers by the gateway. Otherwise, the smart card shares the registration information $V_{i}$; meanwhile, the terminal of the user (e.g., personal computer, laptop) initializes a pair of RSA parameters ($e_{i},d_{i}$), where “$e_{i}$” is the public key and “$d_{i}$” is the private key, and selects a random value $r_{u}\in Z_{p}^{*}$—some body area sensor node $MS_{j}$—with the identifier, $MIS_{j}$, which the $U_{i}$ needs to acquire, and extracts the time stamp $T_{1}$. Then, it computes the following values: $B_{1}=h\left(V_{i}\right)\left|\right|e_{i}⊕h(r_{u}\left|\right|T_{1}),B_{2}=MIS_{j}⊕h(PID_{i}||h(r_{u}\left|\right|T_{1}))$. Next, the verifier obtains $B_{3}=h(PID_{i}||MIS_{j}||h(r_{u}\left|\right|T_{1})||e_{i})$. In the end, the $U_{i}$ sends the request packet $\left\{PID_{i},B_{1},B_{2},B_{3},T_{1}\right\}$ to the GWN in the open channel. It is worth noting that, in computing $B_{1}$, to keep the ’⊕’ operation running properly, the size of $h\left(V_{i}\right)\left|\right|e_{i}$ is equal to the size of $h(r_{u}||T_{1})$; through this, 0 is added in the upper part of $h(r_{u}||T_{1})$.

### 4.4. Verification Phase of the User, the GWN, and the BASNs

In the verification phase, all three entities—the $U_{i}$, the GWN, and the $MS_{j}$—will verify each other’s identities, and then the $U_{i}$ and the $MS_{j}$ negotiate a session key, SK, to protect secret information in future communications.

- **Step 1.** $GWN⟶MS_{j}:\left\{B_{4},B_{5},B_{6},T_{2}\right\}$. Given the login response from the $U_{i}$, the GWN first checks whether $|T_{c}-T_{1}|<\Delta T$; here, $T_{c}$ and ΔT are the current timestamp and the time gap, respectively. If so, then the GWN computes $V_{i}^{*}=h(PID_{i}||x),h(r_{u}^{*}\left|\right|T_{1})||e_{i}^{*}=B_{1}⊕h\left(V_{i}^{*}\right),MIS_{j}^{*}=B_{2}⊕h(PID_{i}||h(r_{u}^{*}\left|\right|T_{1}))$. The GWN checks whether $MIS_{j}^{*}\in L_{revoke}$. If so, then the authentication request for $MIS_{j}^{*}\left(i.e.,MIS_{j}\right)$ is not valid, and the GWN neglects this login request. Otherwise, the GWN further computes $B_{3}^{*}=h(PID_{i}||MIS_{j}^{*}||h(r_{u}^{*}\left|\right|T_{1})||e_{i}^{*})$. Furthermore, the GWN checks whether $B_{3}^{*}=B_{3}$; if not, then this session concludes. Otherwise, the GWN selects a nonce or a random value $r_{g}\in Z_{p}^{*}$, extracts the timestamp $T_{2}$, and then obtains: $x_{j}=h(MIS_{j}||x),B_{4}=h(x_{j}||MIS_{j})⊕e_{i}\left|\right|r_{g}||h(r_{u}\left|\right|T_{1})$ and $B_{5}=MIS_{j}||h\left(V_{i}\right)⊕h(x_{j}\left|\right|r_{g}),B_{6}=h(h(r_{u}\left|\right|T_{1})||r_{g}\left|\right|x_{j}||MIS_{j}\left|\right|T_{2})$. Then, the GWN sends the information packet $\left\{B_{4},B_{5},B_{6},T_{2}\right\}$ to $MS_{j}$ in the open channel.
- **Step 2.** $MS_{j}⟶GWN$:$\left\{B_{7},B_{8},B_{9},B_{1}0,T_{3}\right\}$. Through the request from the GWN, the body area sensor node $MS_{j}$ first checks whether $|T_{c}-T_{2}|<\Delta T$; if not, then this session is concluded. Otherwise, $MS_{j}$ obtains: $e_{i}^{*}\left|\right|r_{g}^{*}||h(r_{u}^{*}\left|\right|T_{1})=B_{4}⊕h(x_{j}||MIS_{j}),MIS_{j}^{*}||h\left(V_{i}^{*}\right)=B_{5}⊕h(x_{j}\left|\right|r_{g}^{*})$. Then, it further computes $B_{6}^{*}=h(h(r_{u}^{*}\left|\right|T_{1})||r_{g}^{*}\left|\right|x_{j}||MIS_{j}^{*}\left|\right|T_{2})$. Next, $MS_{j}$ checks whether $B_{6}^{*}=B_{6}$; if not, then this session is concluded. Otherwise, $MS_{j}$ chooses a nonce $r_{s}\in Z_{p}^{*}$, extracts timestamp $T_{3}$, and computes $r_{s}^{\prime }={\left(r_{s}\right)}^{e_{i}}$ and $SK=h(h(r_{u}\left|\right|T_{1})||r_{s}||h\left(V_{i}\right))$, $B_{7}=MIS_{j}⊕h\left(r_{g}\right)$, $B_{8}=r_{s}^{\prime }\left||h(SK|\right|r_{g})⊕x_{j}$, $B_{9}=h(r_{s}^{\prime }\left||h(SK|\right|r_{g})||x_{j})||T_{3})$. Next, the verifier obtains $B_{10}=h(SK||r_{g})⊕x_{j}⊕h(r_{s}^{\prime }\left||SK\right)$. Finally, $MS_{j}$ sends the information packet $\left\{B_{7},B_{8},B_{9},B_{10},T_{3}\right\}$ to the GWN in the open channel.
- **Step 3.** $GWN⟶U_{i}:\left\{B_{11},B_{12},B_{13}\right\}$. Upon obtaining $B_{7},B_{8},B_{9},B_{10},T_{3}$, the GWN first checks whether $|T_{c}-T_{3}|<\Delta T$; if not, then this session is concluded. Otherwise, the GWN computes $MIS_{j}^{*}=B_{7}⊕h\left(r_{g}\right)$ and checks whether $MIS_{j}^{*}\in L_{revoke}$. If so, then this denotes that the authentication session from $MIS_{j}^{*}\left(i.e.,MIS_{j}\right)$ is not valid, and the GWN neglects this request. Otherwise, the GWN further computes $x_{j}^{*}=h(MIS_{j}^{*}||x),r_{s}^{\prime *}||h(SK^{*}\left|\right|r_{g}^{*})=B_{8}⊕x_{j}^{*}$ and $B_{9}^{*}=h(r_{s}^{\prime *}||h(SK^{*}\left|\right|r_{g}^{*})||x_{j}^{*})||T_{3})$, and checks whether $B_{9}^{*}=B_{9}$; if so, then the GWN further computes $h(r_{s}^{\prime }\left||SK\right)=B_{10}⊕h(SK||r_{g})⊕x_{j}$ and then selects a new pseudo-identifier $PID_{i}^{new}$, computes $V_{1}^{new}=h(PID_{i}^{new}||x),B_{11}=V_{i}^{new}⊕V_{i}$, and $B_{12}=PID_{i}^{new}\left|\right|r_{s}^{\prime }⊕h(V_{i}^{new}||h(r_{u}\left|\right|T_{1})),B_{13}=h(V_{i}^{new}||h(r_{s}^{\prime }\left||SK)\right)$. Next, the GWN transmits $\left\{B_{11},B_{12},B_{13}\right\}$ to the physician, $U_{i}$.
- **Step 4.** When receiving feedback from the GWN, the $U_{i}$ computes $V_{i}^{new*}=B_{11}⊕V_{i},PID_{i}^{new*}\left|\right|r_{s}^{\prime }*=B_{12}⊕h(V_{i}^{new*}||h(r_{u}\left|\right|T_{1})),r_{s}^{*}={\left(r_{s}^{\prime }*\right)}^{d_{i}}$ with a private key, $d_{i}$; then, $SK^{*}=h(h(r_{u}\left|\right|T_{1})||r_{s}^{*}||h\left(V_{i}\right)),B_{13}^{*}=h(B_{1}^{new*}||h(r_{s}^{\prime *}||SK^{*}))$. After that, the $U_{i}$ checks whether $B_{13}^{*}=B_{13}$; if so, then the $U_{i}$ accepts $SK^{*}$ as SK. Furthermore, they update $A_{1}^{new}=V_{i}^{new}⊕HPW_{i},A_{2}^{new}=h(ID_{i}||PW_{i}\left|\right|V_{i}^{new}\left|\right|V_{ii})$ mod $n_{0}$. Finally, the $U_{i}$ replaces the smart card’s old parameters $\left\{PID_{i},A_{1},A_{2}\right\}$ with newly $\left\{PID_{i}^{new},A_{1}^{new},A_{2}^{new}\right\}$.

### 4.5. User Password Change Phase

The password change phase enables the user to update their password at will. Specifically, it consists of two parts: user identity verification, finished by the smart card; update parameters covering $PW_{i},A_{1},A_{2}$, finished by the user. That is, the $U_{i}$ only submits their old or frequently used password to the smart card, as shown in the login phase. After the smart card verifies the $U_{i}$’s legitimacy—through checking whether $A_{2}^{*}=A_{2}$—the smart card allows the $U_{i}$ to choose a new $PW_{i}^{new}$, and updates the $HPW_{i}^{new}=h(ID_{i}||PW_{i}^{new})$ mod $n_{0},A_{1}^{new}=HPW_{i}^{new}⊕V_{i},A_{2}^{new}=h(ID_{i}||PW_{i}^{new}\left|\right|V_{i}\left|\right|V_{ii})$ mod $n_{0}$. Lastly, the smart card replaces $A_{1},A_{2}$ with $A_{1}^{new},A_{2}^{new}$.

### 4.6. Body Area Sensor Node Deletion Phase

Given that some nodes may be compromised or run out of their limited energy, let us take $MS_{j}$; at this time, the GWN directly revokes this sensor node and puts $MS_{j}$’s identity $MIS_{j}$ into a revocation list $L_{revoke}$. Lastly, the GWN broadcasts $L_{revoke}$ to all communication entities within the WBANs.

### 4.7. Body Area Sensor Node Addition Phase

In this part, the proposed protocol offers a dynamic node addition phase to meet the real-time data collection persistently from the patient. When a new $MS_{t}$ needs to be added into the existing architecture, the GWN only assigns an identifier $MIS_{t}$ and computes $x_{t}=h(MIS_{t}\left||x\right)$ to $MS_{t}$. Then, the new body area sensor node $MS_{t}$ stores the corresponding $x_{t}$ in its secure memory.

## 5. Analyses of the Proposed Protocol

Here, we provide analyses of the proposal, including a security analysis and a performance analysis. The security analysis involves a provable proof security and a heuristic analysis, which shows that our scheme can be robust. Then, the performance analysis includes comparisons of our designed scheme with other new WBAN-oriented schemes, to indicate that the proposed protocol can be applied in real-world uses.

### 5.1. Formal Security Analysis of The Proposed Protocol

As an effective method to prove the semantic security of the protocol, the formal security analysis covers two aspects. That is, given the adversary model shown in Section 3, we need to (1) firstly provide some introductions for formal proof and then state the security objectives of the protocol in Section 5.2; (2) second, in Section 5.3, we provide Theorem 1 to determine the advantages of adversary breaking for the session key in the protocol.

### 5.2. Introductions for Formal Proof

In the proposed protocol **P**, three participants (a physician—$U_{i}$; a gateway node—GWN; body area sensor node—$MS_{j}$) are involved. Initially, the simulator uses the RSA encryption and decryption algorithm over two large primes p,q, where |p|=|q|. Next, the $U_{i}$ obtains their own information $\left\{ID_{i},PW_{i},Bio_{i}\right\}$ and smart card containing $\left\{PID_{i},BKG\left(\cdot \right),A_{1},A_{2},Cou\right\}$; the GWN generates a long-term key *x*; the $MS_{j}$ keeps the identity secret key pair $MIS_{j},x_{j}$.

During the proof, the three entities will instantiate $U_{i}$, GWN, and $MIS_{j}$ with $\begin{array}{c} \prod_{u_{i}}^{u},\prod_{GWN}^{g}, \end{array}$$\begin{array}{c} \prod_{MS_{j}}^{m} \end{array}$, respectively. Furthermore, these instances can be uniformly marked as $\prod_{}^{t}$ if there is no need to tell the three instances apart. Furthermore, if the input message is valid/incorrect or null, then the state of the instance as an oracle will reach accept/reject, or return “⊥”, which means that there is no response for the input.

Here, we provide some terms used in this proof.

- *Accepted state*: When an instance $\prod_{}^{t}$ receives the last expected protocol message, an instance $\prod_{}^{t}$ obtains an accepted state. In this session, all ordered concatenation communicated messages decide on the session identifier.
- *Partnering*: Here, mutually authenticated $\prod_{}^{t_{1}},\prod_{}^{t_{2}}$ are partnering, if $\prod_{}^{t_{1}},\prod_{}^{t_{2}}$ simultaneously satisfy the following criteria: (1) both have an accepted state; (2) both share the same identification; (3) both $\prod_{}^{t_{1}},\prod_{}^{t_{2}}$ are the mutual partners of each other.
- *Adversary*: Based on the information received by initiating the query oracles and controlling the simulator, an adversary A attempts to compromise the security of the authentication messages and rebuild the session key in protocol **P**. Some queries A that can launch are the following:
  - $Execute(\begin{array}{c} \prod_{u_{i}}^{u},\prod_{GWN}^{g},\prod_{MS_{j}}^{m} \end{array})$. This query can be run to simulate the entire authentication process, and A will obtain communicated messages among $U_{i},GWN$ and $MS_{j}$.
  - $Send(\prod_{}^{t},l)$. A can launch an active attack against a participating instance $\prod_{}^{t}$ with a message *l*. Furthermore, if $\prod_{}^{t}$ received the valid *l*, then the simulator gives a response to A. Otherwise, the simulator ends the query.
  - $Reveal(\prod_{}^{t})$. This query means that A can grasp the session key calculated by $\prod_{}^{t}$ (and its partner).
  - $Corrupt(\prod_{u_{i}}^{u},\alpha )$. In this query, A can obtain the corresponding authentication factors stored by the user, $U_{i}$, according to the value α. That is, the oracle exposes the password (α=−1), the data stored in the smart card (α=0), and the biometric information $Bio_{i}\left(\alpha =1\right)$, respectively, to A.
  - Corrupt$\left(\prod_{GWN}^{g}\right)$. For this query, the long-term key *x* could be known by A.
  - Corrupt$\left(\prod_{MS_{j}}^{m}\right)$. A in this query can obtain the secret value of $MS_{j}$.
- *Freshness*: If the session key between the $U_{i}$ and the $MS_{j}$ has not been revealed to A using Reveal, then the instance $\prod_{u_{i}}^{u}$ or $\prod_{GWN}^{g}$, or $\prod_{MS_{j}}^{m}$ can be fresh.
- *Test $\left(\prod_{}^{t}\right)$*: In this test query, A is capable of querying only once. By the protocol **P**, the instance $\prod_{}^{t}$ can, accordingly, only be $\prod_{u_{i}}^{u}$ or $\prod_{MS_{j}}^{m}$. Formally, if instance $\prod_{}^{t}$ has not computed a session key or $\prod_{}^{t}$ cannot be fresh, or $Test(\prod_{}^{t})$ has been queried before, then the test query outputs “⊥” (null). Otherwise, the oracle will flip the unbiased coin *b*. If b=1, the adversary A receives the real session key. If b=0, then A obtains a random string that has the same length as the real session key.
- *Semantic Security*: Given a protocol **P**, a probabilistic polynomial time (PPT) adversary A has requested new instances for a series of queries including the execute query, the send query, the corrupt query, and the test query. Now, A desires to break the protocol **P** by guessing the value of *b* in the test query and outputting a guessing value $b^{*}$. Let Succ(A) denote the event that A guesses $b^{*}$ correctly *b*, i.e., $b^{*}=b$. The advantage of A breaking the semantic security of protocol **P** over the session key can be defined as follows:
  $$Adv_{A}^{P}=2\mathrm{Pr}[Succ(A)]-1.$$

### 5.3. Semantic Security Proof of The Protocol

In this part, we show the proposed protocol’s semantic security evaluation in the view of a theorem.

**Theorem 1.**  *Let P be the proposed protocol, |D| be the space of a password, and n be the system security parameter. After making a series of queries—including execute-query $q_{e}$ times, send query $q_{s}$ times, hash query $q_{h}$ times, and bio-hashing query $q_{BKG(\cdot )}$ times—the advantage $Adv_{A}^{P,D}$ of A breaking the semantic security of SK in P is less than*

$$\frac{q_{h}^{2}+6q_{s}}{2^{l_{1}}}+\frac{{\left(q_{s}+q_{e}\right)}^{2}}{p}+\frac{q_{BKG(\cdot )}^{2}+2q_{BKG(\cdot )}}{2^{l_{2}}}+2\left(C^{\prime }q_{send}^{s^{\prime }}+Adv_{A}^{RSA}\left(n\right)\right)$$

**Proof.**  By the games chain, involving Game_1_–Game_8_, we now prove that the adversary’s advantage in breaking the semantic security of session key is factually negligible. Furthermore, set $Succ_{i}$ to the event in which A successfully guesses the *b* in the test query of Game_*k*_, where k=1,2,⋯,8.

Game_1_: this game simulates a real attack by the random oracle. A bit *b* is then randomly chosen at the beginning of this game. Thus,

(1) $$Adv_{A}^{P,D}=2\mathrm{Pr}\left[{\mathrm{Succ}}_{1}\right]-1$$

Game_**2**_: this game shapes a hash list $\Omega_{h}$ and a BKG(·) list $\Omega_{BKG(\cdot )}$. Say that A initiates a hash query h(γ), then the hash oracle $\Theta_{h}$ takes γ to retrieve $\Omega_{h}$. If a hash value h(γ) is retrieved in $\Omega_{h}$, then $\Theta_{h}$ responds the hash value. Otherwise, a random string ψ will be sent to A; meanwhile, (γ,ψ) is stored in $\Omega_{h}$.

For BKG(·)’s oracle $\Theta_{BKG(\cdot )}$, its simulation is simulated in the same way as the hash oracle $\Theta_{h}$. By the known list in this game, A performs the Test-query to tell the real session key and the random value apart. For $SK=h(h(r_{u}\left|\right|T_{1})||r_{s}||h\left(V_{i}\right))$, secret values only include $U_{i}$’s $r_{u},V_{i}$, and $MS_{j}$’s $r_{s}$. Hence, A has no way to compute SK and to distinguish whether b=0 or b=1 other than to guess.

Thus, compared to Game_1_, A’s chance of winning Game_2_ does not increase the A’s advantage despite its eavesdropping attack, i.e.,

(2) $$\mathrm{Pr}\left[{\mathrm{Succ}}_{2}\right]=\mathrm{Pr}\left[{\mathrm{Succ}}_{1}\right]$$

Game_**3**_: In this game, A can execute an active send query or hash query to try to persuade a communication entity to accept a forged message. Compared with Game_1_ and Game_2_, A’s advantage may be enhanced by finding the collision to generate a valid message. That is, if the following collisions occur, then this game aborts.

(i) A collision can be found in the hash values or BKG(·)’s outputs, and the probability is $\frac{q_{h}^{2}}{2^{l_{1}+1}}$ or $\frac{q_{BKG(\cdot )}^{2}}{2^{l_{2}+1}}$, where $l_{1}$ and $l_{2}$ denote the length of the output by the hash function and BKG(·), respectively.
(ii) Another collision which can be found is on the choice of random numbers $r_{u},r_{g},r_{s}$; the probability is $\frac{{\left(q_{s}+q_{e}\right)}^{2}}{2p}$.

Thus, we have:

(3) $$|\mathrm{Pr}\left[{\mathrm{Succ}}_{3}\right]-\mathrm{Pr}\left[{\mathrm{Succ}}_{2}\right]|⩽\frac{q_{h}^{2}}{2^{l_{1}+1}}+\frac{q_{\mathrm{BKG}(\cdot )}^{2}}{2^{l_{2}+1}}+\frac{{\left(q_{s}+q_{e}\right)}^{2}}{2p}$$

Game_**4**_: In this game, A wants to guess $B_{3},B_{6},B_{9},B_{13}$ without asking the hash query. Obviously, we obtain:

(4) $$|\mathrm{Pr}\left[{\mathrm{Succ}}_{4}\right]-\mathrm{Pr}\left[{\mathrm{Succ}}_{3}\right]|⩽\frac{q_{s}}{2^{l_{1}}}$$

Game_**5**_: In this game, A tries to guess $A_{2}$ without asking the hash query. Similarly, we can obtain:

(5) $$|\mathrm{Pr}\left[{\mathrm{Succ}}_{5}\right]-\mathrm{Pr}\left[{\mathrm{Succ}}_{4}\right]|⩽\frac{q_{s}}{2^{l_{1}}}$$

Game_**6**_: In this game, by the corrupt ($\prod_{u_{i}}^{u},\alpha$) query, A computes $A_{1}$. There are three cases we need to consider.

- Case 1, i.e., corrupt ($\prod_{u_{i}}^{u},\alpha =-1,0$): the probability that A guesses the user’s biometric information is less than $\frac{q_{BKG(\cdot )}}{2^{l_{2}}}$.
- Case 2, i.e., corrupt ($\prod_{u_{i}}^{u},\alpha =1,0$): in the technology of “fuzzy keywords + honeywords”, the probability that A guesses the physician’s password is no more than $C^{\prime }q_{send}^{s^{\prime }}$ [36,37].

Here, $C^{\prime }$ and $s^{\prime }$ are constants, depending on the password dataset, and can be gained through linear regression. Take the Gmail password dataset [38] as an example, $C^{\prime }=0.020963,\;s^{\prime }=0.225653$.

- Case 3, i.e., corrupt ($\prod_{u_{i}}^{u},\alpha =-1,1$): the probability that A guesses values of $A_{1}$ is less than $\frac{q_{s}}{2^{l_{1}}}$.

Therefore, we obtain:

(6) $$|\mathrm{Pr}\left[{\mathrm{Succ}}_{6}\right]-\mathrm{Pr}\left[{\mathrm{Succ}}_{5}\right]|⩽C^{\prime }q_{\mathrm{send}}^{s^{\prime }}+\frac{q_{s}}{2^{l_{1}}}+\frac{q_{\mathrm{BKG}(\cdot )}}{2^{l_{2}}}$$

Game_**7**_: This game describes the attack that A aims to compromise the body area sensor node $MS_{j}$ by performing the corrupt ($\prod_{MS_{j}}^{m}$) oracle, and then A obtains the secret value $x_{j}$ and further $r_{s}^{\prime }$. However, A cannot retrieve $r_{s}$ from $r_{s}^{\prime }$, since there is no PPT solution to break the hardness of large number factorization problem [8]. Therefore, we can yield:

(7) $$|\mathrm{Pr}\left[{\mathrm{Succ}}_{7}\right]-\mathrm{Pr}\left[{\mathrm{Succ}}_{6}\right]|⩽{\mathrm{Adv}}_{A}^{\mathrm{RSA}}\left(n\right)$$

Game_**8**_: In this attack, A tries to calculate SK. At this time, A cannot query the oracle execute query, send query and Corrupt query any more. Similarly to the analysis of Game_7_, A cannot compute $r_{s}$ from $r_{s}^{\prime }$. In other words, A’s advantage in Game_8_ is equal to the advantage in Game_7_. Thus, we can have:

(8) $$\mathrm{Pr}\left[{\mathrm{Succ}}_{8}\right]=\mathrm{Pr}\left[{\mathrm{Succ}}_{7}\right]$$

Until now, we can obtain that A has no non-negligible advantage other than $\frac{1}{2}$, and so $\mathrm{Pr}\left[{\mathrm{Succ}}_{8}\right]=\frac{1}{2}$. From Equations (1)–(8) and triangular inequality, we yield the following deduction with $\Delta =2(C^{\prime }q_{send}^{s^{\prime }}+Adv_{A}^{RSA}\left(n\right)$:

(9) $$\begin{array}{c} \begin{array}{cc} Adv_{A}^{P,D} & =2\mathrm{Pr}[{\mathrm{Succ}}_{1}]-1\\ & =2\mathrm{Pr}\left[{\mathrm{Succ}}_{8}\right]-1+2\left(\mathrm{Pr}\left[{\mathrm{Succ}}_{1}\right]-\mathrm{Pr}\left[{\mathrm{Succ}}_{8}\right]\right)\\ & ⩽\frac{q_{h}^{2}+6q_{s}}{2^{l_{1}}}+\frac{{\left(q_{s}+q_{e}\right)}^{2}}{p}+\frac{q_{BKG}{\left(\cdot \right)}^{2}+2q_{BKG}\left(\cdot \right)}{2^{l_{2}}}+\Delta \end{array} \end{array}$$

As a conclusion, one can see that, if the adversary desires to break the semantic security of a session key, then the advantage of this adversary can only be negligible, $Adv_{A}^{P,D}$, which is less than $\frac{q_{h}^{2}+6q_{s}}{2^{l_{1}}}+\frac{{\left(q_{s}+q_{e}\right)}^{2}}{p}+\frac{q_{BKG}{\left(\cdot \right)}^{2}+2q_{BKG}\left(\cdot \right)}{2^{l_{2}}}+2\left(C^{\prime }q_{send}^{s^{\prime }}+Adv_{A}^{RSA}\left(n\right)\right)$. □

### 5.4. Heuristic Security Analysis of The Proposed Protocol

The heuristic method [7] does not involve any complex formula. It is a very effective and simple method, which can conduct a concise security analysis of the protocol. In this part, our designed protocol provides not only desired attributes, but is also resistant against a variety of known attacks.

- Mutual authentication. The proposed scheme can obtain mutual authentication, since the $U_{i}$ and the GWN authenticate each other bidirectionally by checking whether $B_{3}^{*}=B_{3}$ and $B_{13}^{*}=B_{13}$, respectively. Then, through the $MS_{j}$ checking whether $B_{6}^{*}=B_{6}$ and the GWN seeing that $B_{9}^{*}=B_{9}$, the GWN and the $MS_{j}$ can authenticate each other.
- Session Key Agreement. The session key agreement means that no one can solely pre-compute the session key without interacting with another entity. Factually, in the proposed scheme, $SK=h(h(r_{u}\left|\right|T_{1})||r_{s}||h\left(V_{i}\right))$ contains the indispensable part from the $U_{i}$ (the secret parameter $r_{u}$) and the $MS_{j}$ (the secret parameter $r_{s}$), and so our scheme meets this well-defined attribute.
- Forward Secrecy. Forward secrecy holds if the past built session keys are still secure, on the condition that the long-term secret—i.e., the GWN’s *x*—is corrupted. As a matter of fact, suppose that the attacker knows *x*, and further that they can obtain the $PID_{i}$ from the open channel and then compute $V_{i}=h(PID_{i}\left||x\right)$, and then obtain $h(r_{u}||T_{1})$. Even so, it is vitally important to note that they cannot retrieve the $r_{s}$ because of the hardness of the large number’s factorization in RSA [8]. That is, we can obtain forward secrecy.
- User Anonymity. User anonymity mainly consists of user identity protection that cannot be figured out by the adversary and the user’s un-traceability, which guarantees that the adversary can neither determine who the user is nor distinguish whether two occurrences of data interaction are by the same user. For identity protection, in the registration phase, the $U_{i}$ only submits $A_{0}$ to the GWN, so it does not directly extract the identity information for the adversary, even if the GWN is destroyed. The $PID_{i}$ cannot be used to deduce the identity of a user during the authentication phase, and so the adversary cannot capture the user’s identity $ID_{i}$. As for the un-traceability of the user, the randomness of $PID_{i}$ breaks the statistical property, which effectively confuses the adversary in their attempt to determine whether two data behaviors are from the same entity.
- Password Guessing Attack. There are two password guessing attacks that result from the verification value: one is in a smart card (attack I) and the other is the verification value in a public channel (attack II). For attack I, even if the adversary knows the verification values $A_{1},A_{2}$ in the smart card, they cannot check the correctness of the guessed $PW_{i}^{*}$ and $ID_{i}^{*}$, because of the congruence of the “modulus” operation in $HPW_{i}$ and $A_{2}$. For attack II, the password-related verification value only is attributed to $B_{1}$. Although the adversary obtains $B_{1}$ and even owns $A_{1}$, they cannot verify the correctness of the guessed $PW_{i}^{*}$ and $ID_{i}^{*}$, because the indeterminacy and congruence of the “modulus” operation confuses the adversary in their attempt to decide which of the guessed values ($ID_{i}^{*},PW_{i}^{*}$) is correct [39].

Additionally, according to the research work of [39], the space of the adversary to guess the identity and the password is $\frac{|D_{id}\left|\times \right|D_{pw}|}{n_{0}}$, where $2^{4}\leq n_{0}\leq 2^{8},|D_{pw}\left|=\right|D_{id}|=10^{6}$. So, the valid password and identity cannot be effectively guessed by the adversary, since the $\frac{|D_{id}\left|\times \right|D_{pw}|}{n_{0}}\approx 2^{32}$ is larger than the finite value Cou, which denotes the time data of the smart card, leading to login failure for the adversary. Thus, the proposed protocol is safe against password-guessing attacks.

- Body area sensor node impersonation attack. The adversary in this attack [7] is mainly the legitimate inside user. The user could obtain the body area sensor node’s secret key $x_{j}$, leading to a faulty session key for the next new physician. Factually, this adversary cannot extract this secret $x_{j}$ from $B_{7},B_{8},B_{10}$, since they cannot obtain the value $r_{g}$ of the GWN. So, this attack in the proposed scheme has no favorable space.
- Desynchronization attack. Generally, after the session key is established, $U_{i}$, GWN, and $MS_{j}$ have no need to update any parameters, and so the desynchronization attack is impossible. However, the $U_{i}$ in our scheme needs to change their pseudo-identity $PID_{i}$ to $PID_{i}^{new}$ and $B_{1}$ to $B_{1}^{new}$, and then check whether $h(B_{1}^{new*}||h(r_{s}^{\prime *}||SK^{*}))=B_{13}$. Luckily, it is verifying the correctness of $B_{13}$ that guarantees the synchronization update of $PID_{i}$ and $B_{1}$.
- Replay attack. The adversary in the replay attack usually sends old messages to obtain the verification of the participants. In the proposed protocol, the $U_{i}$, the GWN, and the $MS_{j}$ choose random numbers $r,r_{u}$, $r_{g}$, and $r_{s}$, respectively, to ensure the freshness and independence of the exchanged messages in each session. As a result, the adversary cannot obtain authentication from another through the replay attack.
- Verifier-stolen attack. For an adversary using verifiers to launch an attack, since there is no verifier table associated with the user being stored in the GWN, the verifier-stolen attack cannot occur.
- Privileged insider attack. In this attack, the adversary (even a corrupted GWN) can extract the real or bare identity information of a legitimate user in the registration phase. Factually, the $U_{i}$ just submits an $A_{0}$ that encapsulates the $ID_{i}$ to the GWN, rather than the bare $ID_{i}$. Therefore, the identity of the user can be protected in this attack.
- Node capture attack. This attack denotes that the adversary has the node’s secret value, $x_{j}$, and then retrieves $A_{3}$ and $A_{4}$. However, this adversary cannot re-calculate the session key, SK, unless they can effectively solve the problem of the large number’s factorization.
- Denial of service (DoS) attack. In the proposed scheme, even if the adversary may render BASN unavailable by repeatedly replaying the old message $B_{4},B_{5},B_{6},T_{2}$, the BASN firstly verifies whether the time gap meets $|T_{c}-T_{2}|>\Delta T$ or not. If so, then the BASN directly terminates this session. Furthermore, even though the adversary updates the timestamp $T_{2}$ to make $|T_{c}-T_{2}|<\Delta T$, the BASN also ignores this session, because of the following verification failure of value $B_{6}$, where $B_{6}$ can only be derived by the original timestamp. Thus, this DoS attack makes no sense. Similarly, the terminal of the GWN can resist the DoS attack.
- Man-in-the-middle (MITM) attack. In our protocol, suppose that the adversary [40] listens to and blocks the user’s login message $PID_{i},B_{1},B_{2},B_{3},T_{1}$, the response message $B_{11},B_{12},B_{13}$ from the GWN, and extracts all the parameters of the smart card. To issue a man-in-the-middle (MITM) attack, the adversary must forge another new message flow $PID_{i}^{*},B_{1}^{*},B_{2}^{*},B_{3}^{*},T_{1}^{*},B_{11}^{*},B_{12}^{*},B_{13}^{*}$ or replay the old messages. As discussed above, the proposed scheme can resist an impersonation attack and replay attack. That is, it is not possible for the adversary to be authenticated by both the user and the gateway. Hence, the proposed scheme is resistant against the MITM attack.
- Session-specific temporary information attack. This attack happens if the adversary learns the value of session key by obtaining short-term information like random values or nonces, $r_{u},r_{s}$. However, in our scheme, apart from the nonces, the long-term information like $V_{i}$ constitutes an SK, and so this attack is infeasible for adversaries.

### 5.5. Performance Analyses in Functionality and Consumed Cost

In this section, we provide details on the detailed performance analyses covering the functionality comparisons and cost comparisons among the WBAN-oriented user authentication schemes.

For a long time, indispensable valuable design criteria have been used to effectively evaluate the advantages and disadvantages of extant authentication protocols; meanwhile, these provide guidance for designing a good protocol that obtains a balance between performance and security. In accordance with the new criteria [20] and according to our security analyses shown above, Table 2 describes 10 detailed criteria, comprising five ideal (E_*_) attributes and five security (C_*_) attributes.

Furthermore, in Table 3, for the five ideal attributes, it can be observed that all schemes meet E_2_, i.e., sound repairability. However, a difference appears in the remaining four attributes. Specifically, scheme [31] does not involve the “password” as an authentication factor and so there is no comparison to be made (denoted by ‘**-**’). However, the scheme presented by [28] shows weakness in E_3_, with no secret user or sensor node constituting the session key.

For the five security attributes, the sensor node capture attack threatens the session key’s security, which indicates that [28,31,34] cannot meet C_4_. In the scheme of [28], the user’s password security and the session key’s forward secrecy cannot be guaranteed, since the adversary can easily initiate an effective password guessing attack and compromise the GWN. This implies that the scheme presented in [28] cannot meet C_2_, C_3_, or C_5_. For the schemes presented in [31,34], the GWN can grasp the identities of communication entities, which makes it easy for the attacker to obtain identity values by corrupting the GWN. Accordingly, the works presented in [31,34] do not meet C_1_. Meanwhile, in the scheme presented by [31], no one verifies the identity of the relay node, and so E_4_ cannot be achieved.

Our scheme is thus superior to the alternatives. That is, by using the technology of the RSA algorithm, the dynamic assigning of the pseudo identity, and “the modulus” operation, our scheme successfully fulfills the 10 criteria.

Next, we present a comparison among the consumed overheads, covering the storage–communication–computation costs. To obtain a comprehensive evaluation of the overhead comparisons, Table 4 pre-defines the reasonable reference length of all the terms for the compared schemes [28,31,34].

In Table 5, for the aspect of storage costs consumed, one can see that the user, the gateway, and the BASN in our protocol need 640 bits, 320 bits, and 160 bits, respectively. However, the storage costs of these three entities in other schemes are unavoidably influenced by the following parameters: *N* (the number of challenge–response pairs in [28]); *m* (the number of users), *n* (the number of BASNs), and $m^{\prime }$ (the number of relay nodes) in [31]. Please note that, in [31], from the flow of mutual authentication and key agreement phase in Figure 2 of [31], the role of $SN_{j}$ can be seen as that of the $U_{i}$, the role of MS can be seen as that of the BASN, and the role of RN can be seen as that of the GWN. Thus, more storage costs (i.e., total 128N(2m+n)+640(m+n)+416 in [28] and $128(m^{\prime }+n)+928$ in [31]) will be consumed as the parameters increase. Overall, the proposed protocol is advantageous compared with the compared schemes.

**Figure 4:**
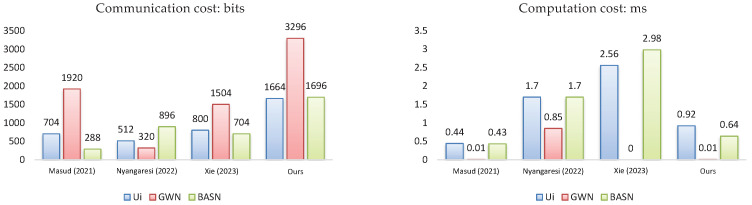
Comparison of communication and computation costs in all schemes [28,31,34].

As for the communication costs comparison shown in Figure 4, it can be seen that our protocol consumes more communication costs than other compared schemes in order to meet all the attributes shown in Table 3; other schemes save in communication costs, but subsequently weaken the security of the authentication protocol.

As for the computation costs, since the login phase and the verification phase are frequently run through a user authentication protocol, we provided the cryptography computation costs of these two phases. Then, by running the test algorithm on compiler CLion (version 2023.2) in the Windows 11 operating system with 12th Intel core i7-12700H, 16G memory, where the compiler was developed by JetBrains, located in Prague, Czech Republic, we determined that the estimated time of the 1024-bit RSA modular exponentiation is 0.63 ms and the time for the scalar multiplication of the ECC is 0.85 ms. Furthermore, for other cryptography functions, the time for the BKG is 0.29 ms [41], the time for the hash function (SHA-1) is 0.00069 ms [42], and the time for the PUF is 0.43 ms [43]. As shown in Figure 4, the time consumption values from the user and the BASN in our scheme were 0.92 ms and 0.64 ms, respectively. These show the potential for reducing the user’s and the BASN’s computation costs by 64% and 79% in comparison with the scheme presented by [31].

In summary, as evidenced by the provable security demonstrated in Section 5.3 and the heuristic analysis demonstrated in Section 5.4, our proposed mechanism can ensure the mutual authentication, forward secrecy of the session key, and user anonymity, while resisting all known attacks. As demonstrated by the performance analyses presented in Section 5.5, we show that the proposed protocol meets the 10 design criteria; other schemes show deficiencies in the provision of the key agreement (E_3_), the mutual authentication (E_4_), and the security criteria from C_1_ to C_5_. Combined with Figure 4 and Table 5, one can see that the costs of storage and computation are superior to the schemes presented by [28,34]. Hence, we can determine that the proposed protocol outperforms the baseline protocols.

## 6. Conclusions

The authentication mechanism has always been an effective method of guaranteeing the security of data sharing for WBANs. In this paper, based on the RSA encryption and decryption algorithm, we propose a robust three-factor authentication protocol for WBANs. Through detailed security proofs and heuristic analyses, we prove that the proposed protocol can resist various known attacks. Finally, the performance analyses were evaluated to show that the costs of storage and computation are superior to the schemes proposed by [28,34]; specifically, our proposal can reduce the user’s and the BASN’s computation costs by 64% and 79%, respectively, compared to the scheme proposed in [31], which indicates that our protocol would be more suitable for WBANs with limited resources. For our future research, we will focus on the authentication of WBANs in the architecture of decentralized identity (DID) through blockchain. 

## Figures and Tables

**Table 1:** Notations of symbols in our scheme.

Symbols	Descriptions	Symbols	Descriptions	Symbols	Descriptions
$U_{i}$	*i*th physician	GID	GWN’s identity	$MS_{j}$	*j*th BASN
$PW_{i}$	Password of $U_{i}$	*x*	GWN’s long-term key	$MIS_{j}$	$MS_{j}$’s identity
⟹	The secure channel	⟶	The public channel	⊕	The XOR operation
$ID_{i}$	Unique identity of $U_{i}$	BKG(·)	Biometric key generation	$x_{j}$	Secret value of $MS_{j}$
$PID_{i}$	Pseudo-identity of $U_{i}$	$bio_{i}$	Biometric information of $U_{i}$	X||Y	The concatenate operation

**Table 2:** Ten criteria for evaluating authentication schemes.

Short-Term				Definition in WBANs
Ideal	Attributes	E_1_	Password friendly	Users are allowed to choose and locally change their passwords at will.
		E_2_	Sound repairability	The BASN can join the network dynamically and the smart card can be revoked.
		E_3_	Key agreement	The user and BASN should and must negotiate a session key after the authentication.
		E_4_	Mutual authentication	All participants should verify each other’s identities.
		E_5_	No password verifier table	Password-related parameters are only stored by the user.
Security	Attributes	C_1_	User anonymity	The users’ identities can neither be calculated nor tracked by the adversary.
		C_2_	No password exposure	In the registration phase, the privileged participants (usually the administer of the gateway) cannot obtain the users’ password.
		C_3_	Forward secrecy	The agreed session key cannot be acquired by A even when the long-term key of gateway is compromised.
		C_4_	Resistance to known attacks	The protocol can resist the impersonation attack, offline guessing attack, desynchronization attack, replay attack, stolen verifier-attack, unknown key share and known key attack, DoS attack, and node capture attack. Note that, in these attacks, A does not compromise the smart card or the BASB anymore.
		C_5_	Resistance to smart card loss attack	A failed to attack the protocol via a user’s smart card.

**Table 3:** Functionality comparisons.

Schemes	Ref.	E_1_	E_2_	E_3_	E_4_	E_5_	C_1_	C_2_	C_3_	C_4_	C_5_
Masud et al. (2021)	[28]	*√*	*√*	*√*	*√*	*√*	*√*	×	×	×	×
Nyangaresi et al. (2022)	[34]	−	*√*	*√*	*√*	−	×	−	*√*	×	*√*
Xie et al. (2023)	[31]	−	*√*	*√*	×	−	×	−	*√*	×	−
Ours	-	*√*	*√*	*√*	*√*	*√*	*√*	*√*	*√*	*√*	*√*

**Table 4:** The length of all terms.

Symbols	Bits	Symbols	Bits
Module $\left(n_{0}\right)$	32	ECC point (p)	160
Counter (c)	32	Hash value (h)	160
Timestamp (T)	32	Secret value (x)	160
Entities’ identity (ID)	128	Random/nonce (r)	160
Challenge–response pair (CRP)	128	Public key of RSA pk	1024
Biometric key generation (BKG(·))	160	Symmetric ciphertext size (enc)	256

**Table 5:** Comparison of storage costs.

Schemes	Ref.	Storage Cost: Bits		
		$U_{i}$	GWN	BASN
Masud et al. (2021)	[28]	288	128N(2m+n)+640(m+n)	128
Nyangaresi et al. (2022)	[34]	640	480	640
Xie et al. (2023)	[31]	$128(m^{\prime }+n)+160$	0	768
Ours	–	640	320	160

## Data Availability

Not applicable.
